# Advancements in hand-drawn chemical structure recognition through an enhanced DECIMER architecture

**DOI:** 10.1186/s13321-024-00872-7

**Published:** 2024-07-05

**Authors:** Kohulan Rajan, Henning Otto Brinkhaus, Achim Zielesny, Christoph Steinbeck

**Affiliations:** 1 Institute for Inorganic and Analytical Chemistry, Friedrich Schiller University Jena, Lessingstr. 8, 07743 Jena, Germany; 2 Institute for Bioinformatics and Chemoinformatics, Westphalian University of Applied Sciences, August-Schmidt-Ring 10, 45665 Recklinghausen, Germany

**Keywords:** Hand-drawn chemical structures, Chemical structure recognition, OCSR, Optical chemical structure recognition, DECIMER, Deep-learning, Transformer

## Abstract

**Abstract:**

Accurate recognition of hand-drawn chemical structures is crucial for digitising hand-written chemical information in traditional laboratory notebooks or facilitating stylus-based structure entry on tablets or smartphones. However, the inherent variability in hand-drawn structures poses challenges for existing Optical Chemical Structure Recognition (OCSR) software. To address this, we present an enhanced Deep lEarning for Chemical ImagE Recognition (DECIMER) architecture that leverages a combination of Convolutional Neural Networks (CNNs) and Transformers to improve the recognition of hand-drawn chemical structures. The model incorporates an EfficientNetV2 CNN encoder that extracts features from hand-drawn images, followed by a Transformer decoder that converts the extracted features into Simplified Molecular Input Line Entry System (SMILES) strings. Our models were trained using synthetic hand-drawn images generated by RanDepict, a tool for depicting chemical structures with different style elements. A benchmark was performed using a real-world dataset of hand-drawn chemical structures to evaluate the model's performance. The results indicate that our improved DECIMER architecture exhibits a significantly enhanced recognition accuracy compared to other approaches.

**Scientific contribution:**

The new DECIMER model presented here refines our previous research efforts and is currently the only open-source model tailored specifically for the recognition of hand-drawn chemical structures. The enhanced model performs better in handling variations in handwriting styles, line thicknesses, and background noise, making it suitable for real-world applications. The DECIMER hand-drawn structure recognition model and its source code have been made available as an open-source package under a permissive license.

**Graphical Abstract:**

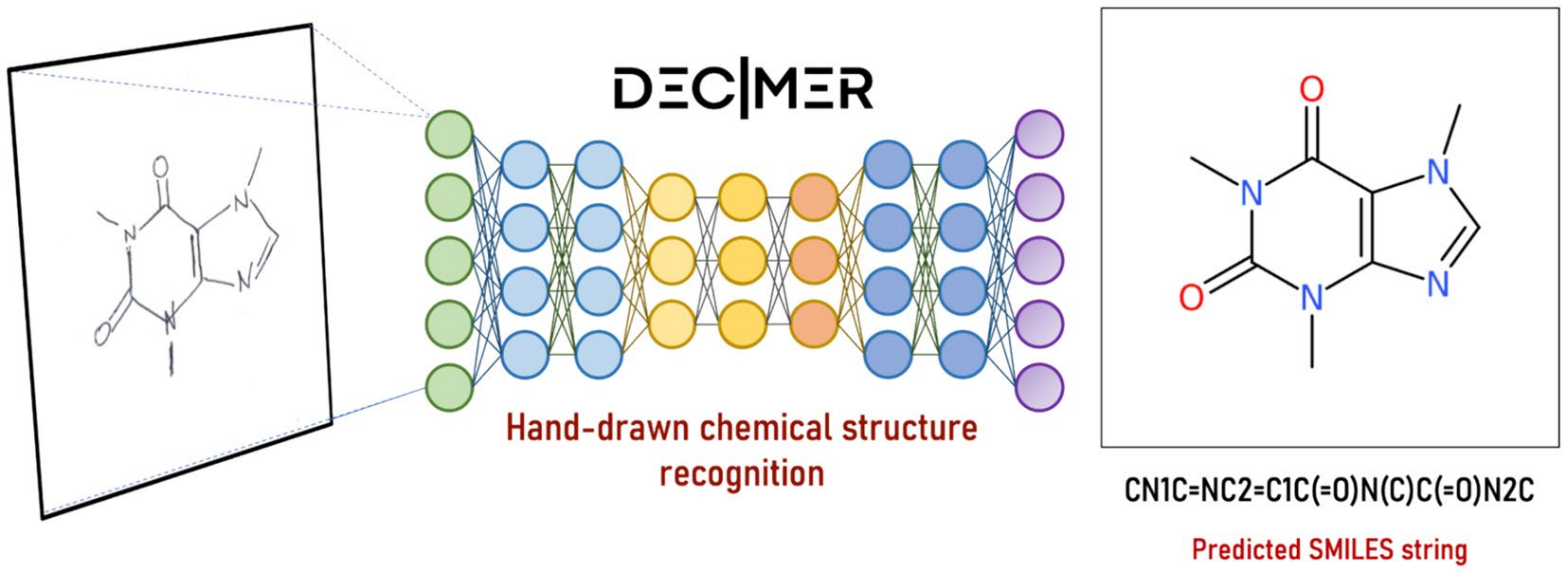

## Introduction

Humans have used hand-drawing and hand-writing for most of our cultural history to create art and capture information. Digitising graphics is common, but capturing their deeper meaning is much more challenging. With the advent of so-called deep learning algorithms, the interpretation of images has seen considerable advances, ranging from the interpretation of medical images to the annotation of personal photo collections.

A key application of deep learning methods in chemistry is mining printed and hand-written documents for information on chemical compounds. Mining of past publications, for example, can augment present open-access databases [1]. While this information can often be found in printed literature, it is typically presented in unstructured, human-readable formats like text and images. Manually curating and organising this information to fill the database gaps is error-prone and time-consuming [2]. Therefore, automation is necessary to improve accuracy and efficiency [3]. A key task is detecting and interpreting chemical structure depictions to translate them into machine-readable formats, commonly called Optical Chemical Structure Recognition (OCSR) [4].

Over the past few years, deep learning methods have been used extensively to conduct OCSR for detecting and converting chemical structure depictions from printed literature [4, 5]. With improvements in computer vision and language models, the field has seen a lot of development [6]. Molecular structures can be represented in images in various ways, using many different drawing styles. When representations of a variety of depiction styles are included in the training data, a data-driven deep-learning approach can be applied to reach a high degree of robustness and flexibility. Rule-based OCSR algorithms that are not based on deep learning have been shown to lack robustness and tend to fail when small distortions are added to the images in common benchmark datasets [7].

In addition to mining chemical information from printed literature, information can also be found in hand-written laboratory notebooks that were never before attempted to be digitised and mined for chemical structure information. In these notebooks, chemical structures are typically manually drawn, which means there is an even higher degree of diversity in how molecular structures are depicted. Unless the chemists choose to publish their novel findings together with related information in a publication, these hand-drawn structures are never converted into machine-readable formats. Recognising and interpreting hand-drawn chemical structures is challenging due to the variety of drawing styles and the complexity of each individual's handwriting [8, 9]. Therefore, it is crucial to develop accurate tools for recognising and digitising hand-drawn chemical structures. Digitising hand-written chemical structures enables high-quality data-driven research and preserves information for future use.

Like hand-written text recognition, hand-drawn chemical structure recognition can be categorised into online and offline recognition tasks [10]. Online chemical structure detection primarily denotes converting a chemical structure drawn on a digital medium, such as a tablet or personal computer, into a machine-readable format in real-time. If the detection is inaccurate, the user can adjust their drawing style to make the system predict the molecule correctly. In contrast, offline chemical structure detection predominantly deals with previously drawn chemical structure images. These images exhibit a wide array of drawing styles, making it considerably more challenging to recognise them with high confidence [11].

Taking these considerations into account, we present an advanced deep-learning method for accurate hand-drawn chemical structure recognition. We introduce an encoder-decoder model that combines the EfficientNetV2 Convolutional Neural Network (CNN) with a Transformer Decoder-only model. This combination aims to identify and transform hand-drawn chemical structures into a machine-readable file format with higher confidence. Our approach builds upon the DECIMER image transformer [6, 12], a deep learning-based OCSR method for extracting chemical structural data from printed literature. There is a growing interest in identifying hand-drawn chemical structure depictions, as this has the potential to streamline the automated digitisation of laboratory notebooks [13].

OCSR methods can be broadly categorized into two main groups: rule-based methods and deep learning-based methods [4]. Rule-based approaches typically involve a systematic sequence of processing steps, including vectorisation, atom detection, bond classification, Optical Character Recognition (OCR) [14], graph compilation, and post-processing. Various rule-based techniques, such as OSRA [15], Imago [16], and MolVec [17], follow a procedure along those lines. In 2021, Clévert et al. showed that the performance of the openly available rule-based systems on commonly used benchmark datasets decreases drastically when slight image distortions are introduced [7]. Apparently, the parameters in the rule-based procedures can be overfit to specific depiction styles and do not necessarily perform well on all types of chemical structure depictions.

In recent years, deep learning-based OCSR methods have become increasingly popular [5], driven by advancements in computer vision and powerful hardware for training complex models. Deep learning approaches excel in processing chemical structure depictions and can effectively process even distorted representations [7]. This capability provides a competitive edge when developing OCSR methods for hand-drawn chemical structures. Since deep learning algorithms can detect more complex patterns, they are an excellent choice for OCSR applications. Additionally, these methods can be trained with large amounts of diverse data, resulting in improved accuracy and reliability. Deep learning methods encompass a range of both closed-source approaches, such as MSE-DUDL [18], MICER [19], Image2SMILES [20], ABC-Net [21], Image-to-Graph Transformers [22], IMG2SMI [23], Molecular-InChI [24], and DeepOCSR [25]. On the other hand, several open-source deep learning algorithms have been published, including ChemGrapher [26], DECIMER Image Transformer [12], ChemPix [11], SwinOCSR [27], Img2Mol [7], MolScribe [28], and MolGrapher [29].

While deep learning methods were initially developed for broad applicability across various types of chemical structure depictions, ChemPix was explicitly designed to recognise hand-drawn chemical structure drawings. One notable constraint of ChemPix is its limited functionality, as it exclusively handles drawings of hydrocarbons and is unsuited for other classes of chemical structure representations. In our recently published study about the DECIMER Image Transformer [6], we provided evidence to show that even though our deep learning model was not explicitly trained on hand-drawn chemical structure representations, it exhibits a (limited) capability to interpret them. Compared with ChemPix, our model can recognise various hand-drawn representations of small molecule structures that go beyond those of hydrocarbons. Furthermore, our findings suggest that the recognition performance of this model could be enhanced by training it on a dataset that contains a wide range of hand-drawn chemical structure images.

This work presents a working solution for translating hand-drawn chemical structures into SMILES representations of the depicted molecules [30]. It was specifically trained using artificial data generated by the open-source structure depiction toolkit RanDepict [31]. Its synthetic hand-drawn feature is capable of producing chemical structure representations that mimic hand-drawn chemical structure drawings [6]. The trained model has been benchmarked against the only available diverse hand-drawn chemical structure dataset, DECIMER hand-drawn images [32]. The approach followed here includes no hard-coded rules and is entirely data-driven. The model has been trained and tested only on openly available data sources.

Using this method, we can achieve recognition performance with high confidence in hand-drawn chemical structure depictions. Furthermore, we improved the accuracy of the recognition results by enhancing the DECIMER Image Transformer model. To determine which encoder-decoder model performs best on the same data set, three different models with different configurations of encoder-decoder architectures have been investigated in this study. Subsequently, the best-performing model was trained on datasets of hand-drawn-like chemical structure depictions of four different sizes generated using RanDepict. Finally, the best-trained model was benchmarked against other deep learning-based OCSR methods using a hand-drawn chemical structure dataset. Compared to other openly available OCSR applications, our approach produces better results, with an accuracy of 73.25% and a Tanimoto average of 0.94. This approach can be used to develop accurate and robust OCSR pipelines for real-world applications. Our hand-drawn chemical structure detection model, which we call the *DECIMER hand-drawn model,* has been incorporated into the DECIMER module and made publicly available. These resources are provided under permissive licenses and accompanied by comprehensive documentation.

## Methods

**Fig. 1:**
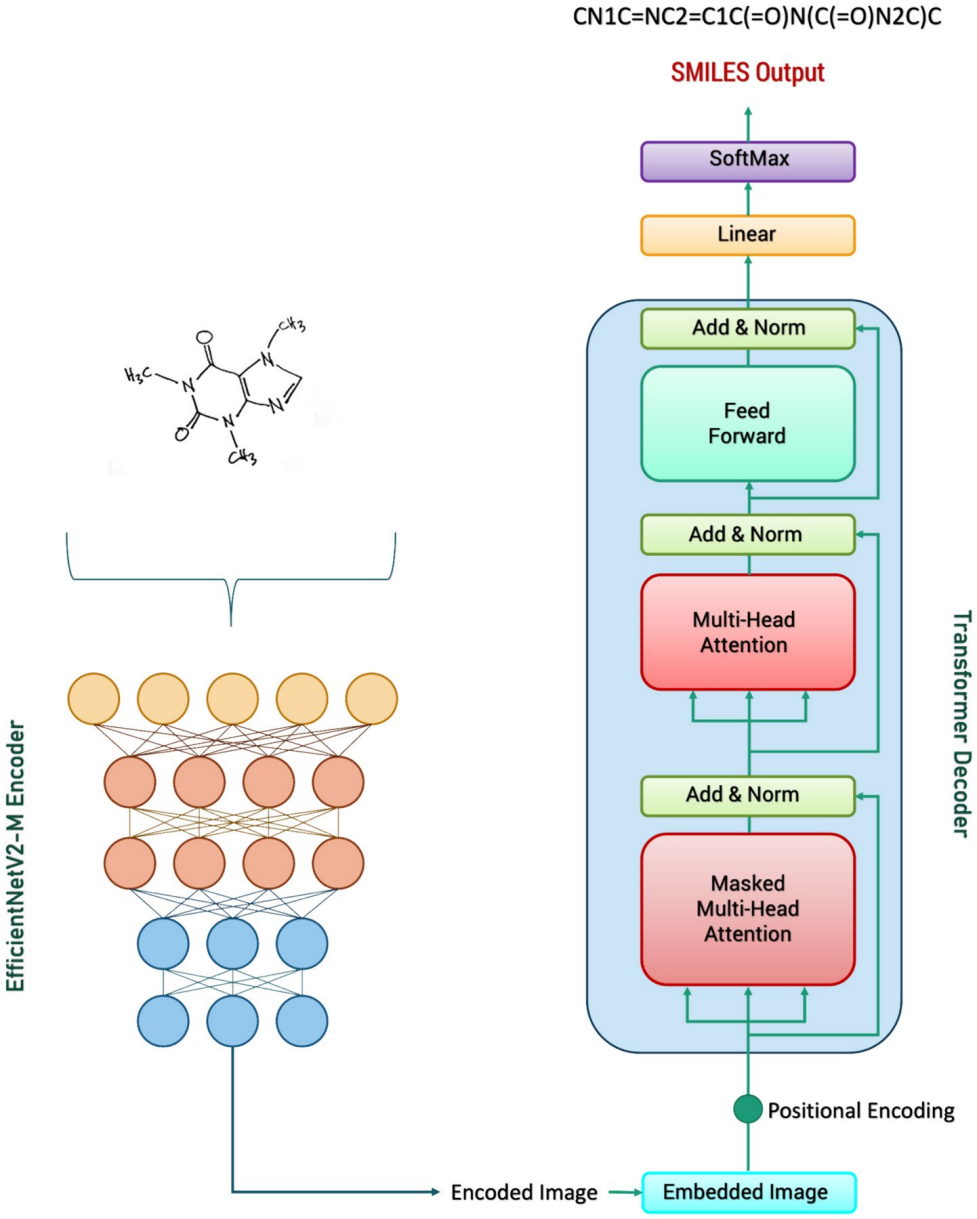
DECIMER hand-drawn chemical structure recognition OCSR model

Here, we introduce an improved version of the DECIMER model designed to recognise hand-drawn chemical structures. The model's architecture is illustrated in Fig. 1. The final model consists of an EfficientNetV2-M encoder combined with a Transformer Decoder, specifically utilising only the decoder component of the transformer. We employed the EfficientNet-V2 M model as a feature extractor by excluding the final fully connected layer and utilising the features generated by the last convolutional layer. The dimensionality of the encoder output is (256, 512), which means it has a spatial dimension of 16 × 16 and 512 channels. This spatial feature map is reshaped into a sequence of length 256, where each element is a 512-dimensional vector. The reshaped encoder output serves as the input to the transformer decoder. The transformer decoder generates an output sequence token-by-token, attending to the encoded image features and the previously generated tokens. Through this process, the encoder analyses the chemical structure images to generate a 2-dimensional feature vector, which the decoder subsequently transforms into a SMILES string.

### Model selection

This work presents an analysis of three different encoder-decoder models. All models feature a CNN encoder based on EfficientNet and a decoder based on the Transformer model [33]. The first model uses the original implementation from our recent publication [6]. It contains an EfficientNetV2-M [34] model as an encoder and a Transformer model as a decoder. The second model uses an EfficientNetV1-B7 [35] encoder and a Transformer decoder. For the third model, EfficientNetV2-M was used as the encoder. In models 2 and 3, only the decoder part of the Transformer model was utilised, while model 1 uses the complete Transformer model. The Transformer models have six decoder layers, eight attention heads, and an embedding dimension of 512 parameters. A detailed summary of these models can be seen in Table 1. All three models were implemented using Python and TensorFlow. The best-performing model was selected as the final model (see Table 1).

**Table 1:** Configurations of the three tested DECIMER Image Transformer models

Model ID	Encoder		Decoder		Batch size	Epochs	Average training time per epoch
	Type	Architecture	Type	Architecture			
1	EfficientNet-V2	M	Transformer	Encoder-Decoder	512	25	36 min
2	EfficientNet-V1	B7	Transformer	Decoder only	512	25	57 min
3	EfficientNet-V2	M	Transformer	Decoder only	512	25	34 min

### Training the models

In this study, we trained all our models on the Google Cloud Platform using the latest Tensor Processing Units (TPUs)—V4. TPUs were selected for this study based on our prior experience, which demonstrated significantly faster training times when compared to in-house Graphical Processing Units (GPUs). TensorFlow was the backend framework, leveraging the TensorFlow distributed training Application Programming Interface (API). The TPU V4 has enabled us to train larger models with more extensive training datasets, yielding improved results. Moreover, TPUs are more energy-efficient than GPUs, facilitating more effective resource utilisation during training.

### Testing the models

The initial models were tested using common OCSR benchmark datasets to determine which model performed best. It was then subjected to further testing later on (see below). The models were primarily evaluated for their ability to recognise chemical structure depictions accurately. This evaluation was based on two key metrics. First, we conducted a one-to-one string comparison using Canonical SMILES for both the original and predicted SMILES representations. This analysis provided insight into how effectively each model predicts chemical structures from input images of chemical structure depictions, with even a single character mismatch in the predicted SMILES string considered as an incorrect prediction.

Additionally, a Tanimoto [40] similarity calculation was performed using PubChem fingerprints, employing the Chemistry Development Kit (CDK) [41] implementation, to compare the original and predicted molecular structures. This approach helped to assess the similarity between the predicted chemical structure and the original one, even when the model's SMILES prediction was inaccurate. This method is particularly valuable because not all predicted molecules precisely match the original, and a quantitative measure aids in understanding the model's performance in interpreting chemical structure depictions. As a result, this comprehensive evaluation approach enhances our understanding of the model's generalisation capabilities.

### Datasets

This section discusses the data sources and the generation of images and textual molecular representations for the datasets used for training the models.

#### Selection of molecules for the datasets

For training and testing models 1 to 3, the latest ChEMBL-32 database was utilised. ChEMBL [42] database version 32 was acquired in the SDF (Structure-Data File) format. The dataset was processed using the CDK SMILES parser functionality to generate canonical SMILES representations preserving stereochemical information. These SMILES strings and their corresponding ChEMBL IDs were then stored in a text file. After analysing the frequency distribution of the length of the SMILES strings, those exceeding 300 characters were removed to eliminate rare, longer SMILES strings. The resulting dataset consisted of a total of 2,290,069 SMILES strings. The RDKit [43] implementation of the MaxMin algorithm [44] was used to select the training and validation datasets. This algorithm enables the selection of diverse data points for both the training and validation data sets. Consequently, the validation set can encompass a chemical space that closely aligns with the training dataset, which will result in a thorough evaluation. This resulted in training and a test dataset of 2,187,669 and 102,400 molecules, respectively. From the resulting training dataset, a subset of 1,024,000 molecules were picked for training the models in this experiment. These were used to train models 1 to 3 and later determine which model was suitable for further experiments.

Similarly, the whole PubChem [45] dataset was processed to select nearly 100 million molecules for training and 100,000 data points for validation during training. Subsets of data were later used to train and test the best-performing model for hand-written structure recognition from this dataset.

#### Training dataset generation

Various chemical structure depictions of the selected SMILES strings were generated using the RanDepict toolkit [31]. The images were created with a resolution of 512 × 512 pixels per image. Each data point was represented by two 8-bit PNG images—one with and one without any image augmentations, excluding hand-drawn-like augmentations. The purpose of introducing augmentation on the images is to mimic real-world scanned pages and to add more complexity. The models were trained using a dataset consisting of 2,048,000 images. These generated images were used as the input for the encoder, and the SMILES strings were defined as the desired decoder output. The SMILES strings were split into meaningful tokens using the Keras tokenizer. The resulting tokenisation scheme splits the input after heavy atoms (such as "C" and "O"), open and closed brackets (such as "(" and ")"), bond symbols (" = " and "#"), special characters(“.”, “-”,” + ”,”\”,”/”,”@”,”%” and”*”), as well as after every single-digit number. A start token " < start > " and an end token " < end > " were added to the beginning and end of each sequence, respectively. Each tokenised string was also padded using " < pad > " tokens.

The generated images with their corresponding tokenised SMILES strings were combined and converted into small chunks of TFRecord files of about 100 MB each. They were then moved to a Google Cloud bucket for training. Datasets were converted into TFRecord files primarily for training on Google Cloud using Tensor Processing Units (TPUs).

**Fig. 2:**
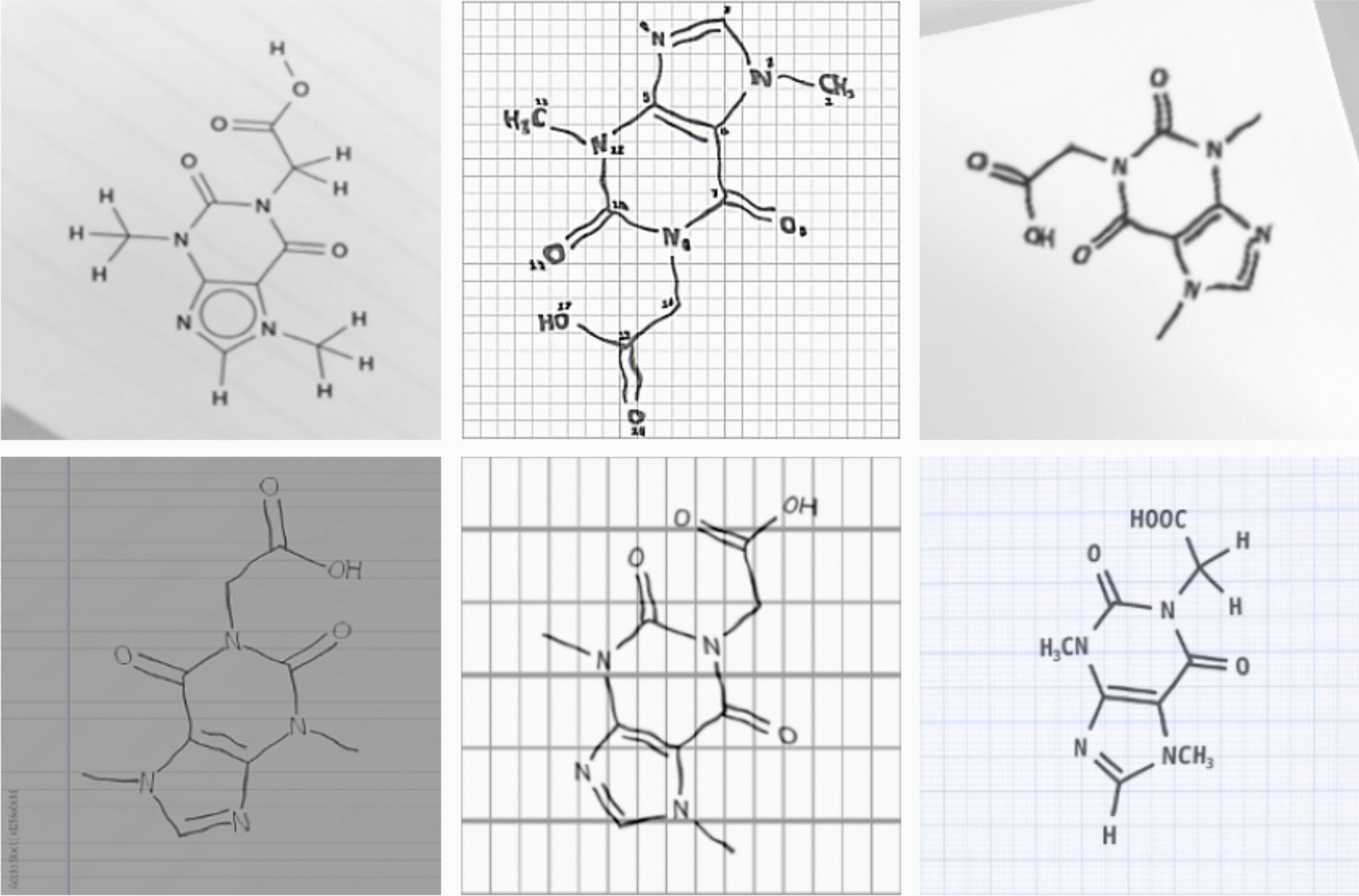
Examples of hand-drawn-like synthetic chemical structure depictions created for the Caffeine molecule through the use of RanDepict

Similarly, the PubChem dataset was used to generate the training dataset for the final model. Using the selected SMILES strings, hand-drawn-like synthetic chemical structure depictions were generated using RanDepict (see Fig. 2). Again, the image size was set to 512 × 512, and the generated data and the tokenised SMILES were saved into TFRecord files and moved to a Google Cloud bucket for training. Here, every molecule was depicted three times without augmentations and once with augmentations.

#### Training datasets

##### Training dataset to train different model architectures

For training different model architectures and selecting the best one, a subset of 1 million data points was randomly selected from the curated ChEMBL database, as mentioned in the section on molecule selection for the datasets. This subset of 1 million data points was used to train various combinations of model architectures and identify the best-performing model through an evaluation process.

##### Training datasets for DECIMER-Hand drawn model

Two training datasets were generated from each ChEMBL and PubChem molecular structure datasets to further train the finalised model. Table 2 summarises the dataset sizes and the number of images with and without augmentations.

**Table 2:** Training dataset summary

Dataset ID	Database	No. of molecules	No of images Without augmentations	No of images with augmentations	Total number of images
1	ChEMBL	2,187,669	2,187,669	2,187,669	4,375,338
2	ChEMBL	2,187,669	8,750,676	4,375,338	13,126,014
3	PubChem	9,510,000	28,530,000	9,510,000	38,040,000
4	PubChem	38,040,000	114,120,000	38,040,000	152,160,000

Datasets 1 and 2 are derived from the ChEMBL database and contain the same number of molecules (2,187,669). These molecules were selected as mentioned above. The difference between training datasets 1 and 2 is the number of images generated for each molecule. Dataset 1 has one image with and one image without augmentations per molecule, while Dataset 2 has four images without augmentations and two images with augmentations per molecule.

Datasets 3 and 4 are derived from the PubChem database. Subsets were filtered out using the MaxMin algorithm from the nearly 100 million molecules in the PubChem dataset. For Dataset 3, a subset of 9,510,000 molecules was selected. For Dataset 4, a larger subset of 38,040,000 molecules was selected, which also incorporates all the molecules from Dataset 3. For each molecule in Datasets 3 and 4, three images without augmentations and one with augmentations were generated.

There was no change in the number of molecules between datasets 1 and 2; however, there was a notable increase in the number of images depicted using each molecule. During the transition from Dataset 2 to Dataset 3, both the quantity of molecules and the number of depictions grew. Furthermore, as the number of molecules expanded from Dataset 3 to Dataset 4, there was a corresponding increase in the volume of depicted images.

#### Testing datasets

The OCSR benchmark datasets were used to test the different model architectures in our first experiment. These are listed below,

- JPO: a set of 450 chemical structure images from the Japanese Patent Office [36]
- CLEF: a set of 992 chemical structure images from the Conference and Labs of the Evaluation Forum test set [37, 38]
- USPTO: a set of 5719 chemical structure depictions from the US Patent Office [36]
- UOB: the dataset of 5740 chemical structure depictions compiled by the University of Birmingham [39]

As part of testing the finalised model and assessing whether a model can improve with increasing dataset size, the DECIMER-Hand drawn images benchmark dataset [32] was used.

#### Training implementation of DECIMER-Hand Drawn model

The models were trained using TensorFlow version 2.13.0. After the initial experiment, the final model was the Model 3 implementation. It consisted of an encoder with an EfficientNetV2-M model using default configurations and a transformer decoder with 6 layers (refer to Fig. 1). These models underwent training for 25 epochs on a TPU V4-128 pod slice. Training employed focal loss and the Adam optimizer, complemented by a custom schedule for the learning rate, as specified in the original transformer paper [33]. A dropout rate of 0.1 was also used. To ensure compatibility with the encoder's settings, the images were preprocessed to attain a size of 512 × 512 before being fed into the encoder.

## Results and discussion

This section analyses the three models we first selected to identify which model architecture yields the best results on all benchmark datasets. Subsequently, the best-performing model architecture was selected for the next experiment to determine whether the model's accuracy could be improved with more training data.

### Testing different model architectures

The performance of the three models on real-world images was evaluated using the OCSR benchmark datasets listed under testing the models. The model performance is presented in Table 3, with '**P**' representing the percentage of identical predictions and '**T**' denoting the average Tanimoto similarity calculated across all structures in a dataset. This table serves as the basis for determining the best-performing model, which was considered a candidate for subsequent stages of the experiment.

**Table 3:** DECIMER Image Transformer model performance on OCSR benchmark datasets compared by identical predictions (P) and Tanimoto similarity (T)

	JPO		CLEF		USPTO		UOB		Average	
	P (%)	T	P (%)	T	P (%)	T	P (%)	T	P (%)	T
Model 1	47.78	0.86	62.00	0.94	56.78	0.95	78.55	0.97	61.28	0.93
Model 2	**64.00**	**0.94**	60.58	0.94	60.29	0.97	86.17	0.98	67.76	0.96
Model 3	62.67	**0.94**	**63.51**	**0.95**	**64.01**	**0.97**	**86.88**	**0.99**	**69.27**	**0.96**

The highlighted results in bold specify the best performing result in each benchmark dataset

Model 1's performance is poorer than Models 2 and 3: apparently, the usage of the entire Transformer model as a decoder leads to a reduction in performance compared to the decoder part of the Transformer architecture alone. By using only the Transformer decoder for decoding and removing the encoder part of the transformer, we achieved much better performance on all the OCSR benchmark datasets. Model 3 slightly outperforms Model 2. This is due to using EfficientNetV1 in Model 2, whereas Model 3 uses an updated architecture, EfficientNetV2. In general image recognition tasks, EfficientNetV2 outperforms EfficientNetV1 [34]. Additionally, due to the compact architecture of EfficientNet-V2, Model 3 could train approximately 2 times faster than Model 2 (see Table 1). After assessing the performance metrics and the training times, the model architecture of Model 3 was picked for further experiments.

### Improvement in model prediction with increasing dataset size

Here, the improvement of the accuracy of the model predictions with an increase in the training dataset size and the introduction of hand-drawn-like images in the training data was assessed. With the hand-drawn-like structure depictions, the complexity of the representations of the chemical structures was increased compared to the previously used clean depictions.

In this part of the experiment, we used the molecule datasets based on ChEMBL and PubChem described under methods in datasets. All of the images used for training the models in this experiment were generated by RanDepict, which generated synthetic hand-drawn images for training the models. The models were then tested on a dataset of real-world images to assess their performance. The DECIMER—Hand-drawn images dataset [16], was used to evaluate the models' performance. The dataset consists of 5088 chemical structure drawings sketched by 23 volunteers. The drawings reflect a wide range of drawing styles. The dataset helps us to understand better how well the model that has been exclusively trained on artificially generated training data performs on real hand-drawn chemical structure images.

### Performance on Hand-drawn dataset

After training each model, it was tested against the DECIMER hand-drawn chemical structure images dataset for accuracy and similarity. The number of valid predictions, i.e. the returned SMILES string was syntactically valid and could be parsed into a molecular structure, is also measured. Every generated SMILES string is validated by parsing it through the CDK SMILES parser. If the parsing process fails, the SMILES string is marked as invalid. Table 4 provides the final average values for overall predictions by comparing each predicted structure with the original structure.

**Table 4:** Model performance with increasing dataset size against benchmark dataset

Model ID	Dataset ID	Total number of images	Percentage of valid predictions (%)	Model accuracy (%)	Average Tanimoto similarity
1	1	4,375,338	96.21	5.09	0.490
2	2	13,126,014	97.41	26.08	0.690
3	3	38,040,000	99.67	70.34	0.939
4	4	152,160,000	99.72	73.25	0.942

As expected, there is a significant improvement in performance by tripling the amount of training data from Model 1 via Model 2 to Model 3, reaching a high percentage of valid predictions above 99%, a substantial accuracy of about 70%, and an average Tanimoto similarity of 0.93, indicating similar input and output structures. However, the next quadrupling of the training data for Model 4 only leads to a slight improvement in performance compared to Model 3, suggesting that the potential of the selected training data has been exhausted and that in the future the diversity of the training data needs to be increased to address the weaknesses of the model specifically.

### Performance comparison with other available methods

The final best model's performance on the DECIMER Hand-Drawn Molecules dataset was compared with other available open-source OCSR methods. The tools were evaluated and compared by executing them on real-world hand-drawn images from the DECIMER Hand-Drawn dataset to provide valuable insights into the applicability of the available tools for processing real hand-drawn structure depictions. The summarised results of these comparisons are presented in Table 5. Our study incorporates both rule-based and deep-learning methods.

**Table 5:** DECIMER model performance compared with all available open-source methods

OCSR tool	Method	Percentage of valid predictions (%)	Model accuracy (%)	Average Tanimoto similarity
OSRA [15]	Rule-based	54.66	0.57	0.17
Imago [16]	Rule-based	43.14	2.99	0.22
MolVec [17]	Rule-based	71.86	1.30	0.23
ChemGrapher [26]	Deep Learning	69.56	N/A	0.09
Img2Mol [7]	Deep Learning	98.96	5.25	0.52
SwinOCSR [27]	Deep Learning	97.37	5.11	0.64
MolScribe [28]	Deep Learning	95.66	7.65	0.59
MolGrapher [29]	Deep Learning	99.94	10.81	0.51
DECIMER.ai [6]	Deep Learning	96.07	26.98	0.69
DECIMER	Deep Learning	99.72	73.25	0.94

As can be seen from the above results, the DECIMER model performs much better overall than other deep learning models. According to the results, the rule-based methods perform significantly worse than all the currently available deep learning methods. It is primarily due to the handcrafted rules that were developed for chemical structure representations found in printed literature, as when we deployed them on a hand-drawn dataset, they were not able to function properly since they are not as flexible as the deep learning tools when it comes to processing hand-drawn chemical structures. While deep learning models tend to display a higher level of robustness on this dataset, the number of valid predictions generated by these models is significantly higher than those generated by rule-based methods since deep learning models are likely to pick up on patterns, contexts and subtleties in the hand-drawn structures since they are more robust to noise and variability because they learn the patterns directly from the training data rather than having hardcoded rules. As a result, they can take advantage of a lot more contextual data in the input to make predictions.

## Conclusion

This study introduces an enhanced encoder-decoder model designed to recognise hand-drawn chemical structures. Leveraging recent advancements in computer vision and natural language processing, our model demonstrates significantly improved accuracy, particularly when trained on extensive datasets which contain synthetic hand-drawn images generated using RanDepict. Comparative analysis with already available open-source methods exhibits highly competitive performance when converting hand-drawn chemical structure depictions into computer-readable file format.

The DECIMER model for hand-drawn chemical structure recognition is now seamlessly integrated within the DECIMER modules and will soon be available to use in the Decimer.ai platform. By providing both the model and its source code to the broader public, we intend to make a substantial contribution to the field of chemical data mining. Furthermore, it will facilitate the development of innovative applications and tools for extracting valuable information from laboratory notebooks.

## Data Availability

DECIMER Image Transformer was developed using data obtained from ChEMBL and PubChem: PubChem: https://ftp.ncbi.nlm.nih.gov/pubchem/Compound/Extras/CID-SMILES.gz. ChEMBL: https://ftp.ebi.ac.uk/pub/databases/chembl/ChEMBLdb/releases/chembl_32/chembl_32.sdf.gz. Code availability: https://github.com/Kohulan/DECIMER-Image_Transformer. Model availability: 10.5281/zenodo.10781330. PyPi Package: https://pypi.org/project/decimer/.

## Abbreviations

ABC-Net: Atom and Bond Center Network
API: Application Programming Interface
CDK: Chemistry Development Kit
CLEF: Conference and Labs of the Evaluation Forum
CNN: Convolutional Neural Networks
DECIMER: Deep lEarning for Chemical ImagE Recognition
JPO: Japanese Patent Office
IMG2SMI: Image to SMILES
InChI: International Chemical Identifier
MICER: Molecular Image CaptionER
MSE-DUDL: Molecular Structure Extraction from Documents Using Deep Learning
OCR: Optical Character Recognition
OCSR: Optical chemical structure recognition
OSRA: Optical structure recognition application
PC: Personal computer
PNG: Portable Network Graphics
SDF: Structure-data file
SMILES: Simplified Molecular Input Line Entry System
TFRecord: TensorFlow Record
TPU: Tensor Processing Unit
UOB: University of Birmingham
USPTO: United States Patent Office
V: Version
